# A constant risk for familial breast cancer? A population-based family study

**DOI:** 10.1186/bcr2260

**Published:** 2009-05-20

**Authors:** Kamila Czene, Marie Reilly, Per Hall, Mikael Hartman

**Affiliations:** 1 Department of Medical Epidemiology and Biostatistics, Karolinska Institute, Nobelsväg 12 A, SE-171 77, Stockholm, Sweden; 2 Department of Epidemiology and Public Health and Department of Surgery, National University of Singapore, 16 Medical Drive, Singapore 117597

## Abstract

**Introduction:**

The incidence of breast cancer in the unaffected breast of women with previous breast malignancy remains constant after the first diagnosis. We investigated whether there is a similar pattern in the breast cancer incidence in first-degree relatives of breast cancer patients. We studied the risk for breast cancer in mothers at ages older than their daughter's age at diagnosis.

**Methods:**

We analyzed a Swedish population-based cohort with complete family links and calculated incidence rates of breast cancer in mothers of 48,259 daughters diagnosed with breast cancer.

**Results:**

The risk for breast cancer in mothers of breast cancer patients is elevated relative to the background population at all ages. Mothers have an overall incidence of 0.34%/year at ages older than a daughter's age at diagnosis. This rate is not affected to any large extent by the daughter's age at diagnosis. A constant incidence rate of 0.40%/year from age 35 years onward is seen in mothers of breast cancer patients diagnosed before 35 years of age. For mothers of daughters diagnosed at age 35 to 44 years the incidence pattern is less clear, with the rate being stable for approximately 20 years after the daughter's age at diagnosis and rising thereafter. Older age at a daughter's diagnosis (≥ 45 years) appears to confer an age-dependent increase in incidence in the mother.

**Conclusions:**

Incidence of familial breast cancer in first-degree relatives may increase to a high and constant level by a predetermined age that is specific to each family. This phenomenon appears inconsistent with accepted theories of malignant transformation.

## Introduction

Studies of familial aggregation of breast cancer identify a family history of breast cancer as one of the strongest risk factors for the disease [1,2]. Familial risks for female breast cancer have been the subject of numerous epidemiological studies [3-7]. A study that re-analyzed 52 epidemiological studies of familial breast cancer presented summary risk ratios of 1.80 and 2.93 for one and two affected first-degree relatives, respectively [8].

Young age at onset of disease within a family has long been regarded as a particularly strong risk factor for breast cancer [9-11]. Several studies have investigated the familial risk for breast cancer in relation to both the proband's age at diagnosis and the age of the person at risk [3,12]. The results are similar, regardless of whether siblings or mother/daughter pairs are studied, and show that breast cancer risk in first-degree relatives of breast cancer patients decreases with both the age at diagnosis of the affected relative and the age of the person at risk [3,13]. However, it is not known whether young age in affected relatives only serves as a proxy for increased genetic risk or whether it is a unique indicator of age at onset in individual families.

Few studies have assessed the risk for disease on the absolute scale. Peto and coworkers [10] suggested that there is a family-specific age of onset and that the risk for breast cancer is age independent thereafter in the family members. This hypothesis contradicts most theories of malignant transformation, in which an age-dependent accumulation of genetic events is proposed. The study was not population based and had limited statistical precision because of small sample sizes [10]. A similar constant risk with increasing follow up has been observed for contralateral breast cancer [14-16]. This pattern also appears to be present in twin sisters of breast cancer patients, in whom the constant risk was observed in both monozygotic and dizygotic female twin pairs [10,17].

We conducted a population-based study to characterize the rate of breast cancer in first-degree relatives at ages older than the index patient's age at diagnosis. In order to obtain a good age range for our study, we examined the risk for breast cancer in mothers of breast cancer patients.

## Materials and methods

### Data

The Multi-Generation Register includes all Swedish residents born after 1931, who were alive in 1960, and all those born thereafter. It contains links between children and parents through their national registration numbers, which are assigned to all residents in Sweden. The register is updated yearly. During the period from 1961 to 2004 the completeness of the Multi-Generation Register became progressively better, and since 1991 it has been considered complete [18]. Approximately 40% of offspring who died before 1991 do not have links to their mothers.

Information on cancer was obtained from the nationwide Swedish Cancer Register, established in 1958. During the period of our study, the Cancer Register was estimated to be at least 98% complete [19]. For each notified cancer, the Cancer Register records the national registration number, ICD (International Classification of Diseases) code, date of diagnosis and other details. Further record linkages to the nationwide Cause of Death and Total Population Registers allowed complete follow up with regard to vital status and date of death, as well as dates of emigration and immigration.

From a total population cohort comprising about 11 million individuals recorded in the Multi-Generation Register, we identified all women born in Sweden since 1932 with a first primary invasive breast cancer diagnosed during the period from 1961 to 2004. Subsequently, we identified all the mothers of these women, resulting in a total of 48,259 mother/daughter pairs in which the daughter had a breast cancer diagnosis. In cases in which more than one daughter from the same family had breast cancer, we randomly selected one of the mother/daughter pairs for inclusion in the study.

The ethics committees of the Karolinska Institute (Stockholm, Sweden) approved the study.

### Statistical analyses

Age-specific incidence rates in mothers of daughters with breast cancer were calculated using 5-year intervals. Mothers were studied up to age 85 years, and daughters could be studied up to age 73 years (born after 1931). For the analysis of complete follow up, person-years at risk began at the birth of the mother or 1 January 1961, whichever came later, independent of daughter's diagnosis. Mothers were followed only from the age at diagnosis of breast cancer in their daughter in order to estimate the incidence in mothers at ages older than their daughter's age at diagnosis. Person-years at risk ended on the date of diagnosis of a breast cancer, diagnosis of another malignant cancer, death, emigration or the closing date of the study (31 December 2004), whichever came first. Age-specific breast cancer risk in the general Swedish population (used for comparison) was estimated from the incidence of breast cancers in mothers from our database. The 'general population' was selected to be comparable to the mothers with affected daughters in terms of year of birth (1887 to 1961).

Poisson regression was used to model the incidence of breast cancer in mothers for each 5-year age category. Rates were adjusted for calendar period in 5-year intervals. Likelihood ratio was used to obtain the *P *value of the test for linear trends in rates by maternal age categories. To investigate whether there is heterogeneity in linear trends of the rates in mothers that are approaching or have already passed the age at diagnosis of their daughters, we split the exposure time of mothers into two periods depending on whether the current age is younger or older than the age at diagnosis of the daughter. We then added to the previous model an interaction term between maternal age and this new variable (before/after). Likelihood ratio was used to obtain the *P *value of the heterogeneity in the trend estimates. In the same manner we tested heterogeneity in the trend estimates for mothers by age at diagnosis in daughters. All data preparation and analysis was done using the SAS statistical package, version 8.2 or higher (SAS Institute Inc., Cary, NC, USA) [20].

## Results

**Figure 1:**
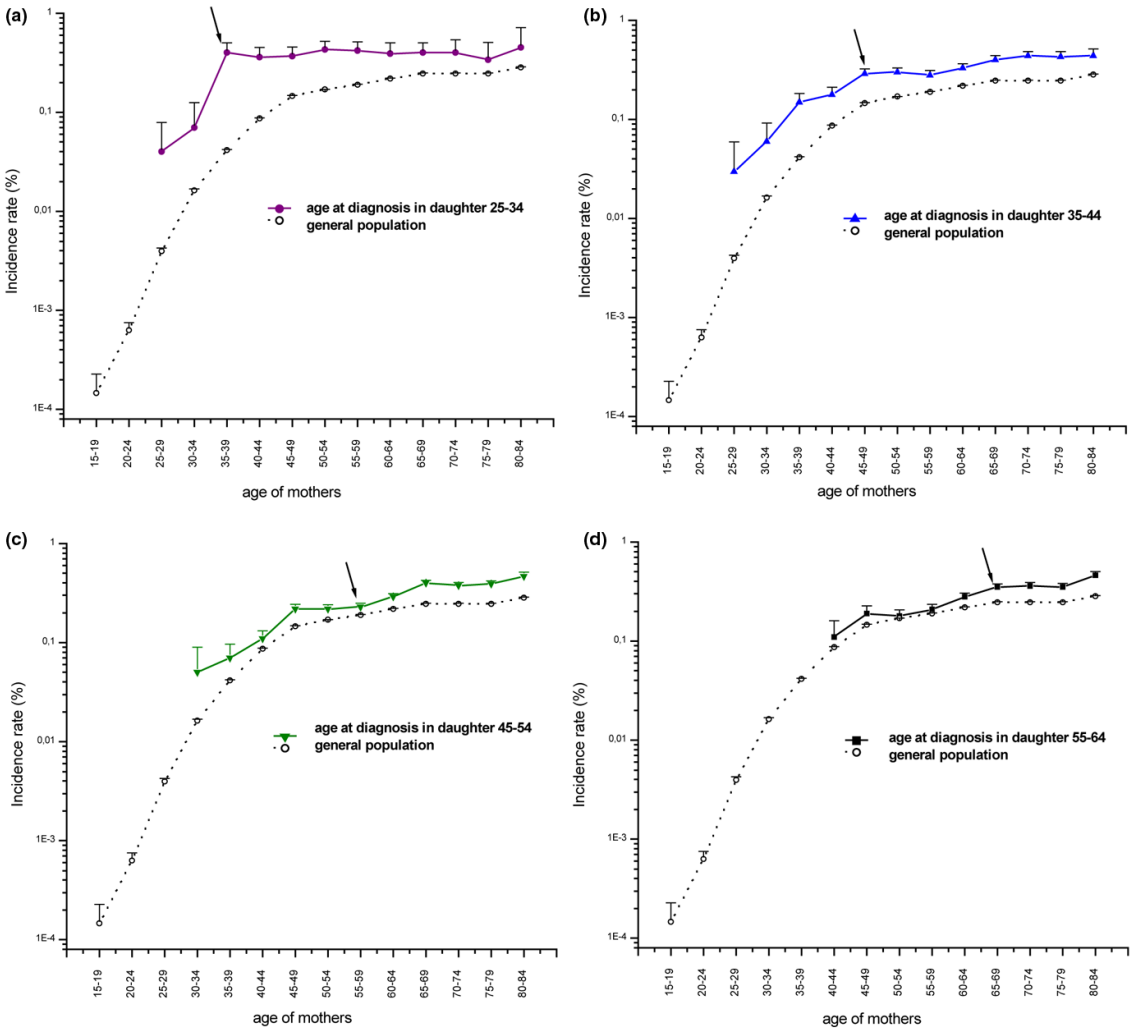
Age-specific incidence rates (with standard errors) of breast cancer in mothers of breast cancer patients. Complete follow-up in mothers stratified by daughter's age at diagnosis: **(a) **25 to 34 years; **(b) **35 to 44 years; **(c) **45 to 54 years; and **(d) **55 to 64 years. Incidence rates of breast cancer in the general population during the period from 1961 to 2004 are included for comparison. Arrows indicate the age group of the mothers that had already attained the age at diagnosis in their daughters.

We identified 48,259 daughters with breast cancer, 3,850 of whom had mothers diagnosed with the same disease. In Figure 1 we present the age-specific incidence rates of breast cancer in mothers stratified by daughter age at diagnosis. The trends in rates of breast cancer had a different pattern in mothers who are approaching and who have already reached the age at diagnosis in their daughter (*P *= 0.0248). Mothers whose daughters were diagnosed before age 35 years had an increasing risk for disease up to the age of diagnosis in the daughter, and the risk remained roughly constant thereafter. Such a trend was not observed for older ages at diagnosis in the daughters. The mothers of daughters with breast cancer continued to be at increased risk (even at ages of 80 to 84 years) compared with women in the general population (Figure 1).

In Table 1 we present the age-specific incidence rates in mothers at ages older than their daughters' age at breast cancer diagnosis. The overall incidence of breast cancer in mothers after the age of diagnosis in the daughters was 0.34%/year. The risk was similar in the strata defined by the daughter's age at diagnosis: 0.40%/year at age <35 years, 0.34%/year at age 35 to 44 years, 0.33%/year at age 45 to 54 years, and 0.35%/year at age 55 years or older.

**Table 1:** Age-specific incidence rates of breast cancer

Age of mothers (years)	Cases (n)	IR (95%CI)^a^
Daughter age at diagnosis <35		
<40	23	0.40 (0.25–0.60)
40–44	23	0.36 (0.23–0.54)
45–49	28	0.38 (0.25–0.55)
50–54	32	0.43 (0.29–0.60)
55–59	30	0.42 (0.28–0.60)
60–64	22	0.42 (0.26–0.64)
65–69	25	0.43 (0.28–0.63)
70–74	14	0.39 (0.21–0.65)
75–79	8	0.34 (0.14–0.64)
80–84	6	0.43 (0.16–0.94)
All ages	211	0.40 (0.35–0.46)
Increase in IR per 5-year age category^b^		0.97 (0.90–1–04)
Test for linear trend^b^		*P *= 0.32

Daughter age at diagnosis 35 to 44		
40–44	21	0.27 (0.19–0.38)
45–49	91	0.29 (0.23–0.36)
50–54	105	0.30 (0.25–0.36)
55–59	100	0.28 (0.22–0.34)
60–64	116	0.33 (0.27–0.40)
65–69	125	0.40 (0.34–0.48)
70–74	108	0.44 (0.36–0.53)
75–79	75	0.43 (0.34–0.54)
80–84	46	0.44 (0.33–0.59)
All ages	787	0.34 (0.32–0.37)
Increase in IR per 5-year age category^b^		1.03 (0.98–1.07)
Test for linear trend^b^		*P *= 0.11

Age at diagnosis in daughters 45–54		
50–54	75	0.23 (0.18–0.29)
55–59	165	0.23 (0.20–0.27)
60–64	212	0.29 (0.25–0.33)
65–69	272	0.40 (0.35–0.45)
70–74	226	0.38 (0.33–0.43)
75–79	181	0.39 (0.33–0.45)
80–84	140	0.47 (0.39–0.55)
All ages	1,271	0.33 (0.31–0.35)
Increase in IR per 5-year age category^b^		1.06 (1.02–1.11)
Test for linear trend^b^		*P *= 0.003

Age at diagnosis in daughters ≥55		
60–64	77	0.25 (0.20–0.31)
65–69	169	0.35 (0.30–0.40)
70–74	184	0.35 (0.30–0.41)
75–79	156	0.35 (0.30–0.41)
80–84	147	0.45 (0.38–0.53)
All ages	733	0.35 (0.33–0.38)
Increase in IR per 5-year age category^b^		1.09 (1.01–1.17)
Test for linear trend^b^		*P *= 0.02

All ages at diagnosis in daughters		
<40	23	0.40 (0.25–0.60)
40–44	44	0.27 (0.20–0.36)
45–49	119	0.31 (0.26–0.37)
50–54	212	0.28 (0.25–0.32)
55–59	295	0.26 (0.23–0.29)
60–64	427	0.30 (0.27–0.33)
65–69	591	0.38 (0.35–0.42)
70–74	532	0.38 (0.35–0.41)
75–79	420	0.38 (0.34–0.42)
80–84	339	0.46 (0.41–0.51)
All ages	3,002	0.34 (0.33–0.36)
Increase in IR per 5-year age category^b^		1.02 (1.00–1.04)
Test for linear trend^b^		*P *= 0.03

Heterogeneity^c^		*P *= 0.0002

Shown are the age-specific incidence rates (IRs) of breast cancer in 48,259 mothers followed after age at diagnosis in daughters in Sweden: 1961 to 2004. ^a^Incidence rate in percentage per year (unadjusted), with 95% confidence intervals (CIs). ^b^Poisson regression model; rates were adjusted for calendar period in 5-year intervals. ^c^Heterogeneity in the trend estimates for mothers by age at diagnosis in daughters.

In our analyses of the age-specific incidence rates in mothers who have already reached the age at diagnosis in their daughters (Table 1), we found evidence of heterogeneity for age at diagnosis in daughters (*P *= 0.0002). Mothers of patients with a breast cancer diagnosis at age under 35 years exhibited a high and constant risk of about 0.40%/year at ages older than their daughters' age at breast cancer diagnosis. No increase in incidence rates by age was seen in this group of mothers, even after adjusting for calendar period using a Poisson model (*P *for trend = 0.32). Mothers with daughters diagnosed at age 35 to 44 years had a risk that appeared to be constant for approximately 20 years after the age at breast cancer diagnosis in their daughter and increased afterward. However, there was no significant difference in trend estimates for these two follow-up intervals (*P *= 0.44). Finally, incidence rates in mothers of daughters diagnosed at age 45 years or older increased steadily with age, with a significant trend (*P *< 0.05) after adjusting for calendar period.

## Discussion

Mothers of breast cancer patients are at increased risk for disease throughout the course of their life in comparison with the background population. At ages older than a daughter's age at diagnosis, her mother has an overall incidence that is not affected to any large extent by the age at diagnosis of her daughter. Mothers of breast cancer patients diagnosed at a young age are at high and constant risk from that age onward. We recognize that it is counterintuitive to consider breast cancer risk in mothers based on future breast cancer diagnosis in their daughters. However, we believe that the two individuals are interchangeable when discussing risk for breast cancer, primarily because the risk in daughters by mother and *vice versa *are similar [3,12]. We also believe that the latent period between their diagnoses does not invalidate the comparison.

The strength of our study is in the prospective population-based design, large sample size, completeness of follow up and unbiased information on family history. Incidence rates for the general population are based on our database and are similar to the rates based on the Swedish Cancer Register [21].

Although it would be more intuitive to study the risk for breast cancer in daughters or sisters of affected women, both of these analyses are subject to serious confounding between age and calendar date because of limitations in the data. Since the index women in our Multi-Generation Register were born after 1932, we could follow daughters only to a maximum age of 73 years, and any cancer in the oldest age group could only be diagnosed during the final years of follow up. As a result, it would be possible to begin follow up in sisters of older women only in recent years, again confounding calendar date and age. By studying the risk in mothers, not only can we follow women at all ages beyond the age at diagnosis of their relative (daughter), but also the diagnoses are evenly spread over the study period, with all ages represented in all years, allowing us to control for confounding by calendar date. Some mothers have been censored from our analyses, because the mothers could not be identified for 40% of women who died before 1991. However, we have shown elsewhere [22] that there is no bias from missing family links, except when there is a very large difference in mortality between familial and nonfamilial cases.

Unilateral breast cancer rates are highly age dependent, being a rare event at young ages and more common as age advances, particularly after the menopause [23,24]. Our analysis of mother/daughter pairs reveals an overall incidence that is apparently independent of the probands' age at diagnosis (0.34%/year). Previous studies of the absolute risk for breast cancer in women stratified by the age of their relative have yielded similar results [10].

The most intriguing finding is the constant risk for breast cancer in mothers who have already attained the age of the index diagnosis in their family. This pattern was seen for mothers of breast cancer patients diagnosed before 35 years of age, with a constant rate for the whole follow-up period (Table 1). For mothers of daughters diagnosed at age 35 to 44 years the pattern was less apparent, with a rate that appeared to be constant for approximately 20 years after the daughter's age at diagnosis and rising thereafter. This pattern was not seen for older age at diagnosis (≥ 45 years) in daughters. The small sample size of the youngest age stratum (daughters <35 years) may result in our having missed a significant trend because of inadequate power. However, the 95% confidence interval (0.90 to 1.04) allows us to exclude confidently an effect of the magnitude found in other age groups and to conclude that the trend – if any – can only be of very small magnitude. One interpretation of our findings could be that in families with a older age at onset the familial cancers are 'diluted', with sporadic cases inducing a less pronounced pattern in terms of age at diagnosis.

An age-independent model of familial breast cancer risk at ages older than the proband's age at diagnosis was previously proposed by Peto and coworkers [10]. Those investigators suggested that the absolute breast cancer risk increases to a high and constant level by a predetermined age that is specific to each family. A similar constant risk with increasing follow up has been observed for contralateral breast cancer, in which the risk for the second cancer is not modified by age and has a constant rate of 0.5%/year [14-16]. This pattern also appears to be present in twin sisters of breast cancer patients; constant risk was observed in both mono and dizygotic female twin pairs [10,17]. This constant hazard could be interpreted as a consequence of an accumulation of a sufficient number of mutations at a certain point in time, resulting in a group with an increased risk for either yet another cancer in the contralateral breast or a first cancer in a first-degree relative. A constant rate for female family members would suggest a similar accumulated predisposition for the disease at a particular unique family-specific age.

The vast majority of familial disease is associated with a low penetrant polygenic aetiology that conveys lifetime risks somewhere below 30%, and only a small proportion are carriers of the highly penetrant mutations of *BRCA1 *or *BRCA2*, in whom the lifetime risks are quite substantial [4,25-28]. Thus far, it has not been possible to establish a risk profile that would warrant prophylactic measures because an insufficient number of these low penetrance variants has been identified. This leaves us with the issue of when to start screening the high-risk populations, especially those who have a relative diagnosed at a young age. Our data indicate a reasonably small risk at ages younger than the proband's age at diagnosis for women with a proband diagnosed at age under 35 years, but we observe a high and constant risk from that age onward. A reasonable interpretation would then be to start screening at least a few years before the proband's age at diagnosis; 5 years before would generate a buffer zone to avoid missed disease in this age category.

## Conclusions

The observed patterns of familial rates of breast cancer suggest that age at onset might be inherited while the sporadic cancers dilute the message by superimposing a background risk in families where cancers are detected at older ages. This phenomenon seems inconsistent with accepted models of susceptibility and has implications for the carcinogenic process of breast cancer. Our findings suggest that screening of unaffected first-degree relatives should start at an age younger than the index patient's age at diagnosis. In the future we will probably be able to identify nonsporadic cancers, and it will become possible to direct screening activities toward these women.

## Competing interests

The authors declare that they have no competing interests.

## Authors' contributions

KC, MH, MR and PH contributed to the design. KC, MH, MR and PH were responsible for the presentation and implementation of this study. KC was responsible for data preparation and conducted the statistical analysis. KC and MH wrote the report. All authors contributed to the interpretation and discussion of the findings and approved the report.
